# Urbanicity and Lyme Disease: 20 Year Trends in Incidence and Case Burden in the United States

**DOI:** 10.1002/puh2.70361

**Published:** 2026-11-06

**Authors:** Madiha Shafquat, L. Hannah Gould, Patrick H. Kelly, Stephanie A. Duench, James H. Stark

**Affiliations:** ^1^ Global Vaccines Medical Affairs Pfizer New York New York USA; ^2^ US Medical Affairs, US Commercial Division Pfizer Collegeville Pennsylvania USA; ^3^ Global Vaccines Medical Affairs Pfizer Cambridge Massachusetts USA

**Keywords:** Lyme disease, public health surveillance, urban health

## Abstract

Incidence of Lyme disease (LD) has increased over the last few decades in the United States (US). We examined trends in LD incidence and case burden in high‐incidence jurisdictions in the US from 2000 to 2021 by urbanicity. County‐level counts of confirmed LD cases from 2000 to 2021 were obtained from national surveillance data. Incidence rates were calculated using census population projections. Urbanicity was categorized using Rural–Urban Continuum Codes (RUCC). Mixed effects models were used to analyze annual changes in LD incidence. Over 70% of LD cases each year were residents of metro counties. All RUCCs had an incidence greater than 10 LD cases per 100,000 per year from 2001 to 2021. LD incidence significantly increased across all RUCCs, with the largest estimated increases in counties in moderately urban areas. Urban populations have substantially contributed to the overall burden of LD in the US, whereas populations in more moderately urban areas had the largest estimated increases in LD incidence. These findings highlight the need for targeted educational interventions and public health measures across urbanicity levels to address the rising burden of LD in the US.

## 1 Introduction

Lyme disease (LD) is the most common vector‐borne disease in the United States (US), with reported incidence of LD increasing over time [1]. Most US LD cases occur in 15 states and the District of Columbia (DC), defined as “high‐incidence” by having an annual incidence of ≥10 surveillance‐reported confirmed LD cases per 100,000 population for three consecutive years [2]. In these areas, humans acquire LD through the bite of an *Ixodes scapularis* tick infected with *Borrelia burgdorferi* [3].

Individual risk of LD results from a combination of acarological hazard (i.e., the presence of biting, infected ticks) and behaviors that result in exposure to that hazard [4]. In recent years, there has been increasing interest in characterizing the acarological hazard in urban and suburban areas, as surveillance has found persistent local transmission in dense urban areas such as Staten Island, NY [5]. Studies in New York City have shown the presence of established *I. scapularis* populations in urban green spaces, likely driven by land use patterns affecting the distribution of host species [6, 7]. A study of LD risk factors found that, though urban residents have lower odds of LD, their risk is more strongly determined by exposure‐related activities [8]. Further, as urbanization increases and the US population residing in metro areas rises [9], fragmentation of areas with infected ticks leads to increased human exposure to this acarological hazard [4].

Understanding the contribution of urban areas to the overall case burden of LD in the US can support messaging and interventions to maximize public health impact. We sought to describe differences in LD case counts and incidence by urbanicity and how these have changed over a 20‐year period.

## 2 Materials and Methods

Counts of confirmed LD cases in high‐incidence jurisdictions (Connecticut, Delaware, DC, Massachusetts, Maryland, Maine, Minnesota, New Hampshire, New Jersey, New York, Pennsylvania, Rhode Island, Virginia, Vermont, West Virginia, Wisconsin) by county of residence from 2000 to 2021 were downloaded from the US Centers for Disease Control and Prevention (CDC) [10]. Data from 2022 to 23 were excluded due to the change in case definition that prevents comparison with historical data [11]. Incidence per 100,000 population per year was calculated using annual US census population projections [12].

Urbanicity was categorized using Rural–Urban Continuum Codes (RUCC), which have shown high concordance with self‐reported urbanicity [13]. RUCC categorizes counties into metro areas (broad labor‐market areas that include central counties with one or more urban areas with populations of 50,000 or more people) and nonmetro areas, and then subcategorizes them on the basis of population size (metro counties) or urban population size and adjacency to a metro area (nonmetro counties) [14]. RUCCs for each county were downloaded from the USDA website for 2003 and 2013, and 2023 [14]. As RUCCs are tied to the decennial census, county codes were applied to the 10‐year census timeframe census; 2003 codes were used for 2000–2009, 2013 codes were used for 2010–2019, and 2023 codes were used for 2020–2021.

Secondary analyses categorized urbanicity using population density per square mile, calculated using annual US census population projections and county land area [12].

Mixed effects negative binomial models were used to calculate the average annual change and 95% confidence interval (CI) in county‐level LD incidence, with fixed effects for the interaction between RUCC category or population density quintile and year and random intercepts for county‐ and state‐level effects. The year × RUCC interaction was used to estimate the annual trend within each RUCC category. Each category‐specific trend was evaluated against no annual change; differences in trends between RUCC categories were not formally tested. Appropriate regression diagnostics were used to confirm validity of model assumptions. Significance was considered a *p* value <0.05 and/or the presence of nonoverlapping CIs. Data from 2020 were excluded from this longitudinal analysis due to the impact of the COVID‐19 pandemic on public health surveillance. All analyses were conducted using R version 4.2.2 [15] with *glmmTMB* [16] and *emmeans* [17] packages.

## 3 Results

From 2000 to 2021, more than 70% of confirmed LD cases reported each year occurred in persons residing in metro counties. Between 42% and 53% of cases each year were reported from metro counties with population sizes of ≥1 million (Figure 1A). Similarly, 60%–80% of confirmed cases reported each year between 2000 and 2021 occurred in persons residing in counties in the top population density quintile (Figure S1A).

**FIGURE 1:**
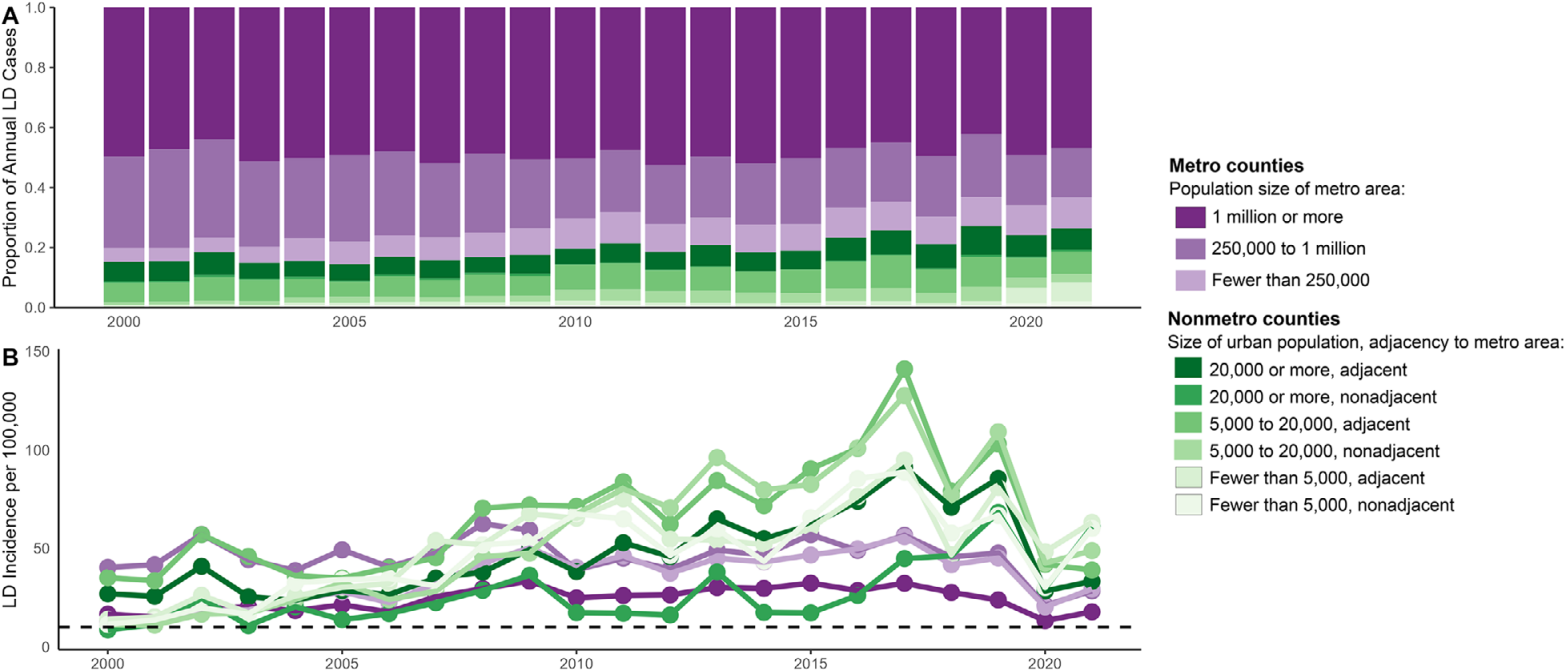
(A) Proportion of Lyme disease cases in high‐incidence jurisdictions, by Rural–Urban Continuum Codes, 2000–2021. (B) Lyme disease incidence per 100,000 population per year in high‐incidence jurisdictions, by Rural–Urban Continuum Codes, 2000–2021. Dashed line at 10 cases per 100,000 population per year.

All RUCC categories had an average incidence of >10 LD cases per 100,000 per year from 2001 to 2021 (Figure 1B). The highest annual county‐level incidence of confirmed LD was in nonmetro counties in all years since 2006 (Figure 1B). Incidence was consistently lowest in counties classified in the highest population density quintile from 2008 to 2021 (Figure S1B). Increases in incidence of LD per 100,000 population per year were greater than 10% in all RUCCs except for metro counties with populations of 1 million or more (Table 1). The largest estimated county‐level increases in incidence were 15.8% and 15.6% per year in nonmetro counties that were nonadjacent to metro areas with populations fewer than 5000 or 20,000 or more, respectively. Overall, counties in all RUCCs had a significant year‐over‐year increase in LD incidence (Table 1). Similarly, all population density quintiles had significant increases in county‐level annual LD incidence, with the third quintile having the highest increase of 1.223 (95% CI: 1.116–1.128) cases per 100,000 population per year (Table S1). Model diagnostics demonstrated moderate overdispersion (dispersion parameter = 1.5), supporting the use of a negative binomial model, and substantial residual geographic clustering (ICC > 0.42) (Table S2).

**TABLE 1:** Average annual change and 95% confidence interval (CI) in county‐level Lyme disease (LD) incidence in high‐incidence jurisdictions from 2000 to 2021^a^, by Rural–Urban Continuum Codes (RUCC).

RUCC	Year‐over‐year incidence rate ratio [95% CI]
1 million or more	1.070 [1.064, 1.075]^*^
250,000–1 million	1.104 [1.096, 1.111]^*^
Fewer than 250,00	1.137 [1.129, 1.146]^*^
20,000 or more, adjacent	1.130 [1.121, 1.140]^*^
20,000 or more, nonadjacent	1.156 [1.135, 1.178]^*^
5000–20,000, adjacent	1.134 [1.126, 1.142]^*^
5000–20,000, nonadjacent	1.131 [1.120, 1.142]^*^
Fewer than 5000, adjacent	1.118 [1.106, 1.130]^*^
Fewer than 5000, nonadjacent	1.158 [1.142, 1.174]^*^

*Note:* Marginal estimates by RUCC are linear combinations of main effect of year and interaction coefficients between year and RUCC level.

^a^2020 data excluded.

*p < 0.05.

## 4 Discussion

The overall burden of LD in the US has increased during the last two decades, and most LD cases reported since 2000 have been in persons residing in highly populated metro areas. These findings underscore that, although local LD transmission in highly urbanized areas may be lower than in rural areas, cases residing in these counties represent a substantial contribution to the overall burden of LD in the US. Furthermore, average annual LD incidence has increased since 2000 in counties across urbanicities. The largest estimated increases in incidence were in suburban areas with populations greater than 20,000 or fewer than 5000, even though suburban areas historically may not have been considered high‐risk LD areas by local populations.

The historical association of LD risk with rural areas [7] is in part driven by the understanding that the presence of infected, biting ticks is a prerequisite for LD risk. Less developed areas with more forest cover have been well‐established as ideal *I. scapularis* habitats [4], whereas developed urban areas with less forest cover are typically less suited for *I. scapularis* survival. Nevertheless, urban areas still incorporate numerous green spaces in the form of parks or yards connected to larger forested areas creating urban‐forest “edges” that harbor substantial *I. scapularis* populations [6, 7]. In addition, these green spaces may also harbor populations of small mammals, which are important reservoirs for *B. burgdorferi* [18, 19]. However, some portion of the LD incidence we see in urban areas may be misattributed as cases are reported to surveillance on the basis of county of residence, which is not necessarily where they acquired the infection. Indeed, a study of surveillance‐reported cases in Minnesota found that LD cases residing in metro areas were more likely to report travel‐related tick exposures than those residing in nonmetro areas [20].

Further, the process of urbanization itself has been identified as a key driver of LD. A study along the I‐95 Northeastern corridor in the US over a 10‐year period found a spatial correlation between urban expansion and LD incidence. In particular, they found that the highest increase in LD incidence was in counties that were urbanizing, rather than statically urban or rural [21]. Our findings extend this conclusion across all high‐incidence jurisdictions: that more moderately urban areas, which have not yet been maximally urbanized, had the largest estimated increases in LD incidence over time. However, the precise mechanism by which urban expansion results in increased LD risk is highly complex, and dependent on aspects related to acarological hazard (e.g., habitat composition, biodiversity, and host movement), human behaviors, and dynamic feedback processes between the two systems [4, 18, 22].

As the population of Americans living in urban areas continues to rise [9], it is important to characterize the role these areas play in LD epidemiology. Although risk of LD is well documented for individuals living in rural areas [8], our findings show that all urbanicity levels had incidences higher than the “high‐incidence” threshold as defined by the CDC of 10 cases per 100,000 population per year [2]. These findings emphasize the need for equitable access to public health prevention and educational tools across all populations at risk.

Our study highlights the significant and rising burden of LD in the US and describes the contribution of urban populations to that burden. Future research may seek to further characterize the mechanisms of how urbanization influences LD risk, and on the development of targeted interventions that address the unique environmental and social dynamics of urban and urbanizing areas.

## Author Contributions

**Madiha Shafquat**: conceptualization, writing – original draft, methodology, visualization, writing – review and editing, software, data curation. **L. Hannah Gould**: conceptualization, supervision, writing – review and editing. **Patrick H. Kelly**: conceptualization, writing – review and editing. **Stephanie A. Duench**: conceptualization, writing – review and editing. **James H. Stark**: conceptualization, supervision, writing – review and editing.

## 5 Funding

This study was supported and jointly funded by Valneva and Pfizer as part of their co‐development of a Lyme disease vaccine. Study design, data analysis, and interpretation of results were conducted independently of vaccine development activities.

## Ethics Statement

The authors have nothing to report.

## Conflicts of Interest

M.S., S.A.D., P.H.K., L.H.G., and J.H.S. are employees of Pfizer and may own stock and stock options in Pfizer.

## Supporting information




**Supporting File**: puh270361‐sup‐0001‐SuppMat.docx.

## Data Availability

The data that support the findings of this study were derived from the following resources available in the public domain—CDC Lyme Disease Data Dashboard, https://www.cdc.gov/lyme/data‐research/facts‐stats/surveillance‐data‐1.html—USDA Rural–Urban Continuum Codes, https://www.ers.usda.gov/data‐products/rural‐urban‐continuum‐codes.
