# Supplementary material for: Urbanicity and Lyme Disease: 20 Year Trends in Incidence and Case Burden in the United States
Source: Public Health Chall. 2026 Nov 6;5(4):e70361. doi: 10.1002/puh2.70361 (PMC13742374; doi:10.1002/puh2.70361)
Supplement: Supplementary file 1 [file PUH2-5-e70361-s001.docx]

**Figure S1: A) Proportion of Lyme disease cases in high-incidence jurisdictions, by population density quintile, 2000-2021. B) Lyme disease incidence per 100,000 population per year in high incidence jurisdictions, by population density quintile, 2000-2021.** Dashed line at 10 cases per 100,000 per year.

**Table S1.** Average annual change and 95% confidence interval (CI) in county-level LD case counts and incidence in high incidence jurisdictions from 2000-2021†, by population density quintile. Marginal estimates by population density quintile are linear combinations of main effect of year and interaction coefficients between year and population density quintile.

| **Population Density Quintile** | **Year-over-year incidence rate ratio [95% CI]** |
| --- | --- |
| <27 persons per square mile | 1.125 [1.116, 1.136] |
| 27-50 persons per square mile | 1.144 [1.136, 1.152] |
| 1.13850-90 persons per square mile | 1.146 [1.138, 1.153] |
| 30-231.1167 persons per square mile | 1.223 [1.116, 1.128] |
| 237+ persons per square mile | 1.075 [1.070, 1.079] |

LD = Lyme disease. *p-value < 0.05. †2020 data excluded.

**Table S2:** Regression model diagnostics and performance statistics.

| **Model Structure (Fixed effects only)** | **Dispersion Parameter** | **Standard deviation [95% CI] of random effect** | | **Intraclass Correlation Coefficient** |
| --- | --- | --- | --- | --- |
|  |  | **State-level** | **County-level** |  |
| Incidence rate ~ RUCC + year + RUCC:year | 1.49 | 2.02 [1.53, 2.67] | 1.05 [1.01, 1.10] | 0.421 |
| Incidence rate ~ Population density quintile + year + Population density quintile:year | 1.50 | 2.05 [1.56, 2.71] | 1.05 [1.00, 1.10] | 0.426 |
